# Engineering strategies for customizing extracellular vesicle uptake in a therapeutic context

**DOI:** 10.1186/s13287-022-02806-2

**Published:** 2022-03-28

**Authors:** Abazar Esmaeili, Mauro Alini, Mohamadreza Baghaban Eslaminejad, Samaneh Hosseini

**Affiliations:** 1 Department of Stem Cells and Developmental Biology, Cell Science Research Center, Royan Institute for Stem Cell Biology and Technology, ACECR, Tehran, Iran; 2 Faculty of Sciences and Advanced Technologies in Biology, University of Science and Culture, Tehran, Iran; 3 AO Research Institute Davos, Davos, Switzerland; 4 Department of Cell Engineering, Cell Science Research Center, Royan Institute for Stem Cell Biology and Technology, ACECR, Tehran, Iran

**Keywords:** Extracellular vesicle engineering, EV-targeting, Customizing EV uptake

## Abstract

Extracellular vesicles (EVs) are advanced therapeutic strategies that can be used to efficiently treat diseases. Promising features of EVs include their innate therapeutic properties and ability to be engineered as targeted drug delivery systems. However, regulation of EV uptake is one challenge of EV therapy that must be overcome to achieve an efficient therapeutic outcome. Numerous efforts to improve the factors that affect EV uptake include the selection of a cell source, cell cultivation procedure, extraction and purification methods, storage, and administration routes. Limitations of rapid clearance, targeted delivery, and off-targeting of EVs are current challenges that must be circumvented. EV engineering can potentially overcome these limitations and provide an ideal therapeutic use for EVs. In this paper, we intend to discuss traditional strategies and their limitations, and then review recent advances in EV engineering that can be used to customize and control EV uptake for future clinical applications.

## Background

It is important to develop new approaches that effectively treat diseases. A promising approach involves the use of extracellular vesicles (EVs). EVs appear to have the potential to accelerate tissue regeneration and improve tissue functions without remarkable side effects. They are small vesicles secreted by cells that are released as exosomes, microvesicles, or apoptotic bodies according to their biogenesis and size. EVs are promising therapeutic options because of the role they play in intercellular communication, their innate therapeutic effects, and their capability to be engineered. EVs are secreted by donor cells and exert their effects after uptake by recipient cells or by releasing their cargo. The use of EVs in clinical settings, in spite of sufficient amounts of effective EV, has encountered difficulties in low rate of EV absorption by their recipient cells, off-target delivery [1], and rapid clearance from circulation [2], which are directly related to EV uptake and could critically affect the EV therapeutic properties. Thus far, many efforts have been made to improve EV uptake by traditional strategies include the selection of an appropriate cell source, cell cultivation procedure, extraction and purification methods, storage, and administration routes [3]. However, limitations to these approaches have urged scientists to engineer EVs in order to improve their therapeutic features and uptake efficiency. The results of these studies have shown that EV's surface cargos greatly affect their uptake **(**Table 1**)** and have a high potential to be engineered [1]. Inner EV cargos could also be engineered to change the targeted recipient cells function [4]. In this paper, we intend to review the traditional strategies in terms of recent advances in improving EVs uptake, and their limitations and challenges. Next, we will discuss EV engineering strategies for customizing EV uptake in order to achieve an efficacious therapeutic outcome.

**Table 1:** Mechanism of extracellular vesicles (EVs) internalization

Route of extracellular vesicle (EV) uptake	EV uptake mechanisms	Interaction factor(s)	Modification/engineering method for targeting	References
Endocytosis				
Clathrin-mediated and Caveolin-dependent	Protein	Tetraspanins	Engineering the CD81 extracellular loop domain on EVs to selectively binds to specific cell surface proteins in donor cells	[72, 118]
		Integrins	Disintegrin inhibitor with specificity for αvβ3 integrin reduce EV uptake in recipient cell	[119]
		Immunoglobulins	By inducing a high-affinity state of leukocyte function-associated antigen-1 on resting T cells to stimulate EV binding	[120]
	Proteoglycan	Heparin sulfate proteoglycans (HSPGs)	A subset of the multiple D-glucosaminyl 3-O-sulfotransferase isoforms prepares binding sites on either the recipient cell surface or EVs	[86]
Lipid raft-mediate	Cholesterol, glycoprotein, protein	Flotillins	Overexpression of flotillins in recipient cell	[121, 122]
Phagocytosis	Protein	C-type lectin	Blocking C-type lectin on the recipient cell surface by specific antibodies to decrease EV uptake	[123, 124]
Macropinocytosis	Protein	Actin	Cytochalasin D hampers actin polymerization and decreases EV uptake in recipient cells	[72, 84]

## Traditional strategies for customizing extracellular vesicle (EV) uptake

In order to exploit the therapeutic potential of EVs, it is necessary to control and adjust EV uptake according to clinical need. Biological, biochemical, and biophysical factors impact EV uptake and they must be adjusted based on the donor and recipient cell types to achieve the desired outcome. The clinical situation determines whether EVs production and uptake need to be increased or reduced by the donor cells. Below, traditional strategies that include donor cell selection, extraction, storage conditions, administration methods and treatment by EVs, recipient cells and their extracellular matrix (ECM) for customizing EV uptake are discussed in detail.

### Control of extracellular vesicle (EV) production by donor cells

Donor cell control of EV production is a basic means to manage EV uptake. Cell source could provide the desired therapeutic effect in EVs such as anti-inflammatory effect, induction of proliferation, special differentiation, etc. [5, 6]. For example, MSC-derived EV [7] and neural stem cell derived EV have been proposed for treatment of osteoarthritis and Alzheimer’s disease, respectively [8]. Selection of the proper cell source and cell culture method enable researchers to control the amount of EVs produced by donor cells and their properties, which greatly impact EV uptake.

#### Extracellular vesicle (EV) sources

EVs isolated from various cell sources could potentially have different innate homing capabilities [9]. EVs from different cellular origins carry different cargos and exert different functions and therapeutic effects on recipient cells [10, 11]. Internal and superficial EV cargos are affected by the content of their cell sources. For example, analysis of RNA contents in donor cells and isolated EVs show similar patterns, which reflect selectivity of the internal cargo packaging into EVs [12]. EV cargo can change during the time that EVs are secreted from the donor cells and taken up by the recipient cells. For example, the presence of microRNA (miRNA)-processing enzymes (e.g., DICER) within the EVs suggests ongoing intravesicular processing that occurs to enable miRNA to mature during transfer and prior to the EV uptake [13]. Superficial cargos play a main role in targeted EV uptake; for instance, cytokines on the EV surface might serve as bar code molecules that are recognized by cell-specific cytokine receptors for targeted EV uptake [14]. Selection of an appropriate cell source enables researchers to improve the quantity and quality of the produced EVs, in addition to the internal and superficial cargos of EVs for targeted uptake. Recent studies demonstrated co-culture of the special cells elevated EV properties for disease treatment [15, 16] due to enriched therapeutic cargos of EVs through prospering interaction between cells. Hence, selection of an appropriate cell/cells source(s) could be enabled researchers to enrich EV and improve their uptake.

#### Amount of secreted extracellular vesicles (EVs)

An inadequate amount of EVs limits their extensive therapeutic use. The amount of secreted EV varies according to its origin [17] and it is also affected by various biological, chemical, and physical factors. Secreted factors like serotonin and histamine, as external biological signals, and microenvironmental conditions that include inflammatory signals can regulate EV release [18, 19]. Environmental factors such as pH and electricity also affect exosome secretion. Acidic pH and low electricity levels increase EV secretion by donor cells without any apparent changes in EV quality [17, 20]. Recent studies have demonstrated that three-dimensional cultures increase the production of EVs and affect their cargo composition [21, 22]. Therefore, regulation of environmental factors for donor cells that include biological, chemical and physical factors, especially in the form of 3D culture platform (such as collagen scaffolds [23] and bioreactor [24]) as cell niche-engineering, could be effective in regulating the quantity and quality of the secreted EVs [7].

### Extracellular vesicle (EV) extraction and purification

EVs of different sizes appear to have different targeting and uptake rates. Recipient cells have been shown to uptake smaller EVs (< 100 nm) at a more rapid rate than larger EVs, which leads to more effective delivery of their cargo and signals [25]. Given the small size of the exosome, they can target tumor tissue via enhanced permeability and retention [26]. Homogeneous populations of EVs would be more safe, stable, and efficient [27]. Therefore, isolation of a monodisperse EV population with a smaller size may improve EV uptake by recipient cells and its subsequent therapeutic effects.

Notably, the reduction of protein contaminants could affect EV uptake. Highly purified EVs appear to have preferential uptake by cells [28]. For example, human endothelial cells uptake EVs isolated from human cardiomyocytes that were highly purified by size-exclusion chromatography (SEC) [28]. EVs isolated by ultracentrifugation, sucrose concentration gradient, SEC, and polymer-based precipitation all differ in yield and purity [29]. SEC has the highest purity among these methods. Although the precipitation method has a lower purity [30], it has a low price and rapid EV extraction and high yield, especially for large scale applications. ExtraPEG is a new polymer-based precipitation that does not affect EV biological activity [31, 32] and leads to smaller particle size distributions and faster uptake by target cells [25]. More recently, heparin-affinity beads have been employed to purify the EVs based on direct EV-heparin interactions. Isolation of EVs from cell culture media and human plasma by ultrafiltration followed by heparin-affinity beads can result in highly pure EVs [33]. These methods should be improved in order to obtain highly purified EVs for preferential uptake in the therapeutic context. In this regard, a combination of several extraction and purification methods would be helpful.

### Extracellular vesicle (EV) storage conditions

It is essential to keep extracted EVs under the best conditions to preserve their therapeutic properties until administration. Storage of EVs has been shown to destabilize the surface characteristics, morphological features, and protein content of isolated exosomes [34]. Particle size decreases with EV storage [35] and affects EV uptake. A review of the literature shows several factors that affect the quantity and quality of EVs during storage and subsequent EV uptake by recipient cells.

#### Temperature

Different storage temperatures and times influence both the recovery yield, morphology, and biological activity of exosomes [35]. Temperatures below − 70 °C are favorable and provide the best conditions for preservation of fresh EVs for clinical applications and basic research [36]. The results of a high throughput study suggested that distinct protein populations leak from exosomes at different storage temperatures [34]. Cheng et al. have evaluated the levels of exosome-associated proteins during long-term storage at different temperatures (− 80 °C, − 20 °C, 4 °C). They observed that ALIX, HSP70, and TSG101 decreased over time and the degradation rate at − 80 °C was less than at − 20 °C and 4 °C [37]. Conversely, in another study, EVs stored at 4 °C had similar stability to those stored at − 70 °C until day 25 [35]. Also, human salivary exosomes remained intact in the absence of protease inhibitor and at different storage temperatures [38]. Although, it is apparent that − 70 °C to − 80 °C is a favorable temperature range for EV storage for clinical use [36], further research is needed to evaluate the impact of temperature and both short-term and long-term storage on EV uptake.

The duration of storage is another factor that might affect EV uptake. Park et al. observed that the numbers of EVs reduced over time; however, this reduction was more noticeable at higher temperatures. EVs stored at − 70 °C for 25 days showed a slight decrease in number [35]. Aggregation is a consequence of EVs stored at − 70 °C and might damage their structure and biological activity [39]. Storage of EVs in a colloidal solution that includes a polymer, such as PEG, is suggested to prevent aggregation of EVs [40] and lead to preservation of biological activity after their uptake.

#### Environmental pH

Acidic conditions are a favorable environment for EV isolation and storage, and might lead to an increase in EV uptake by recipient cells. A pH lower than 7, in conditioned medium or urine, during incubation at room temperature for 30 min has been shown to increase the amount of isolated exosomes [41]. In another study, storage at pH 4 decreased the EV concentration and increased their cellular uptake after 24 h [37].

#### Extracellular vesicles (EVs) and the freeze–thaw cycle

Although the numbers of single exosomes decrease with an increasing freeze–thaw cycle, their cellular uptake is not substantially affected [37]. A decrease in exosome concentration along with an increase in uptake has been reported after 1–5 freeze–thaw cycles and short-term storage (24 h) [37]. In another study, the EV size remained unchanged following multiple freeze–thaw cycles at − 20 °C [42]. Relatively high temperature and freeze–thaw cycles are proposed to affect exosomal membranes and change their properties, which would enable exosomes to be more easily absorbed by recipient cells [37].

According to current research, simultaneous storage of EVs in acidic pH, temperatures between − 70 and − 80 °C, and fewer freeze–thaw cycles would lead to efficacious EV uptake and probably a minimal reduction in EV concentration. However, more research is needed to more accurately determine the factors that contribute to the best EV storage and uptake.

### Extracellular vesicle (EV) administration routes

The type of disease and its progression deeply influence the selection of EV administration strategies. Thus, we can increase EV uptake by using the appropriate EV administration methods and achieve a desired therapeutic outcome. For example, the suggested routes for EV administration to the brain and retina are intranasal (IN) [43] and periocular injection, respectively. Intra-articular injection is recommended for treatment of osteoarthritic joints [38]. However, the administration method may differ for early and advanced stages of cancer. Below, we list the common methods of EV administration for therapeutic purposes.

#### Systemic administration

There is strong preclinical evidence that systemically administered EVs can reach therapeutic tissue targets such as brain [43] or cartilage tissue [5]. Systemically delivered EVs displayed a higher tissue uptake in a positive dose-dependent manner in mice [3] and had a higher chance of reaching the metastatic cells [44]. Moreover, this route of delivery enabled the EVs to be rapidly taken up by macrophages in the reticuloendothelial system and the EVs cleared quickly [45]. The EV half-life is a few minutes and they completely disappear from circulation within four hours after an intravenous (IV) injection [46]. Therefore, the EV circulation time should be increased along with a decrease in clearance in order to maximize their uptake by target cells in order to attain a high therapeutic effect. Next, we discuss the different methods of systemic administration of EVs.

##### Intravenous (IV) injection

IV injection is a common EV administration route for in vivo analysis of EV biodistribution. The results of one study showed that IV injection of EVs might decrease inflammation and apoptosis in an ischemic myocardium [47]. Recent researches showed that IV injections of cardiac progenitor cell-derived exosomes prevented doxorubicin/trastuzumab-induced cardiac toxicity [48]. More than half of the administrated EV remove from the blood within 30 to 60 min after IV injection [49].

##### Subcutaneous (SC) injection

The simplicity of subcutaneous (SC) injection of EVs makes it an ideal route for clinical applications, in particular wound healing. SC injection of exosomes effectively restored epidermal barrier function [50] and attenuated full-thickness skin wounds [6, 51]. In addition, SC injection of exosomes loaded on biological scaffolds promoted diabetic wound healing in a chronic wound [52]. Localization of EVs with biological substrates may increase the efficiency of SC administration of EVs.

##### Intranasal (IN) and inhalation administration

Both IN and inhalation administration are the simplest types of EV administration in the clinic. IN administration of human EVs have been used to treat an injured brain [53]. In another study, IN administration of EVs minimized the adverse effects of status epilepticus in the hippocampus [54]. In addition, the therapeutic efficacy of IN administration of EVs for Parkinson’s disease has been documented [55]. Recently, aerosol inhalation administration of EVs was assessed in various clinical trials as treatment for COVID-19 [56–58]. In this method, EV could suspend in small droplets of liquid (up to a few micrometers in size) and could be absorbed into the respiratory tract and then blood, by spraying using a nebulizer for breathing [59]. This method for EVs administration may soon be the most prevalent method because of its simplicity and ease.

#### Local administration

Local administration in comparison with systemic administration could directly deliver high concentrations of EVs to the site of the injury and increase the ability of recipient cells to uptake EVs. This is particularly relevant when the defect site is enclosed, such as the knee joint space and myocardia [4]. Although local administration reduces off-target delivery of EVs, rapid clearance from the defect site is observed and necessitates repeated administration [60].

The administration route affects uptake of EVs by recipient cells. Therefore, we must select the best administration route for the disease under consideration to enable successful treatment. In general, systemic administration requires a higher total dose for each patient in comparison with local administration [61]. Systemic administration results in a rapid clearance rate of the EVs [45]. In terms of clinical application, the efficiency, simplicity, and cost are important factors that must be taken into consideration when choosing the route of administration.

### Delivery strategies

Taking EVs from isolation to clinical use is an expensive process and finding an optimal situation is required to make most of it. Due to the rapid clearance of EVs from the body, both sustained release and gradual delivery of EVs are expected to increase their efficiency in accordance with the therapeutic goals. Biomaterials, particularly hydrogels, provide an ideal platform for EV delivery in order to enhance their bioavailability, prolong their release, and maximize their regenerative capacity. It was reported that hydrogel-mediated delivery of MSC-derived EVs improved hepatic regeneration in chronic liver failure model [62]. Exosomes loaded on the hydrogel were continually released and promoted chronic diabetic wound healing [52]. Similarly, human umbilical cord (UC)-MSC-derived exosomes encapsulated in functional peptide hydrogels promoted cardiac repair [63]. Advances in tissue engineering, especially hydrogel engineering, and delivery approaches that prolong the existence of EVs in the body would be effective.

### Control of extracellular vesicle (EV) uptake through recipient cells

The amount of EV uptake by recipient cells should be directly regulated in order to achieve an appropriate therapeutic outcome. Disease progression can be halted by either increasing EV absorption or prevention of EV uptake by various means, which include controlling the environmental conditions. For example, uptake and accumulation of human UC-MSC-exosomes by mouse osteosarcoma K7M2 cells in nude mice reduced proliferation and induced apoptosis in the tumors [64]. There are numerous examples for reduction and inhibition of EV absorption to control their uptake.

#### Control of extracellular vesicle (EV) uptake by regulation of the recipient cell environment

Numerous studies have been conducted to investigate the effect of environmental conditions on the rate of EV uptake by recipient cells. EV dose, exposure time, pH, and temperature were assessed. The results indicated a time- and dose-dependent increase in EV internalization under in vitro and in vivo conditions [3, 65–70]. pH can alter EV interactions with cells; therefore, an acidic microenvironment plays a key role in human melanoma progression by increasing EV uptake [20]. Some viral membrane fusion proteins are inactive at pH 7, but undergo conformational changes at pH 5, which leads to membrane fusion during EV uptake [71] and probably after internalization.

It has been shown that when recipient cells are incubated at 4 °C, their capacity to internalize EVs is dramatically reduced compared to incubation at 37 °C [72, 73].

Concurrent control of recipient cells' environmental factors (pH, temperature, dose, and exposure time) regulate EV uptake. However, additional research is necessary to reach more accurate, applicable findings for EV therapy.

#### Control of extracellular vesicle (EV) uptake by treatment strategies

There are various situations in EV therapy where it is necessary to reduce or inhibit its uptake [74] via either direct or indirect strategies. In indirect strategies, drugs such as ketotifen are used to reduce or halt EV secretion by donor cells [75]. Direct strategies are accomplished by treatment of EVs, the recipient cells, and recipient cell ECM components, which we intend to discuss.

##### Direct extracellular vesicle (EV) treatment

EVs secreted by cancer cells or microorganisms could result in disease progression by delivering bioactive molecules, such as proteins and miRNAs to recipient cells. Reduction or inhibition of the uptake of EVs secreted by these cells could be used to treat certain illnesses. For instance, it has been demonstrated that EVs derived from *Helicobacter pylori* preferentially accumulate in the stomach where they induce inflammatory responses [76] and eventually result in stomach cancer. Therefore, we could prevent progression to stomach cancer by either reducing or halting uptake of *Helicobacter pylori* EVs by recipient cells. Targeting superficial EV markers with antibodies or other blocking molecules are presumed to reduce EV absorption. Nishida-Aoki et al. used antibodies against human CD9 and CD63 to disrupt circulating EVs secreted by cancer cells. According to the results, macrophages eliminated the cancer EVs and significantly reduced tumor metastasis [77]. Similarly, treatment of EVs with antibodies against tetraspanin-8, CD49d, integrins, glycans, CD106, and CD11a or CD54 would reduce their uptake [78–81]. Among these, integrins are of utmost importance due to their critical role in EV to cell interactions and EV internalization. EVs secreted by cancer cells assist with intercellular communication between cancer cells during metastasis [74]. Integrin beta 3 (ITGB3) is an EV surface integrin that facilitates EV uptake [82]; hence, peptide blocking of ITGB3 could lead to inhibition of EV uptake and control of metastasis. Targeting the α6β4 and αvβ5 integrins located on the EVs surface by integrin-blocking decoy peptides decreased exosome uptake [83]. Although various EV surface molecules and mechanisms could be employed to control EV uptake by recipient cells, discovering the most potent molecules at the EV surface is not unreasonable and could be promising for EV therapy.

##### Recipient cell treatment

Prevention of EV uptake can also be achieved through blocking recipient cell surface molecules with antibodies, treatment with small molecules, and by mimicking molecules. There was a reduction in EV uptake when monoclonal antibodies on the dendritic cell surfaces blocked integrins α_v_ (CD51) and β_3_ (CD61), CD11a and its ligand CD54, and tetraspanins CD9 and CD81 [80]. Heparin also blocks EV uptake. In another approach, researchers treated cells with a heparin sulfate mimetic molecule, which resulted in a dose-dependent reduction in EV uptake [33, 68]. Recipient cells pre-treated with cholesterol-reducing agents (e.g., filipin) suppressed EV uptake by disrupting lipid raft–mediated endocytosis [70, 72]. A small molecule inhibitor of rac1, NSC23766, also inhibited microglia uptake of EVs [84].

**Fig. 1:**
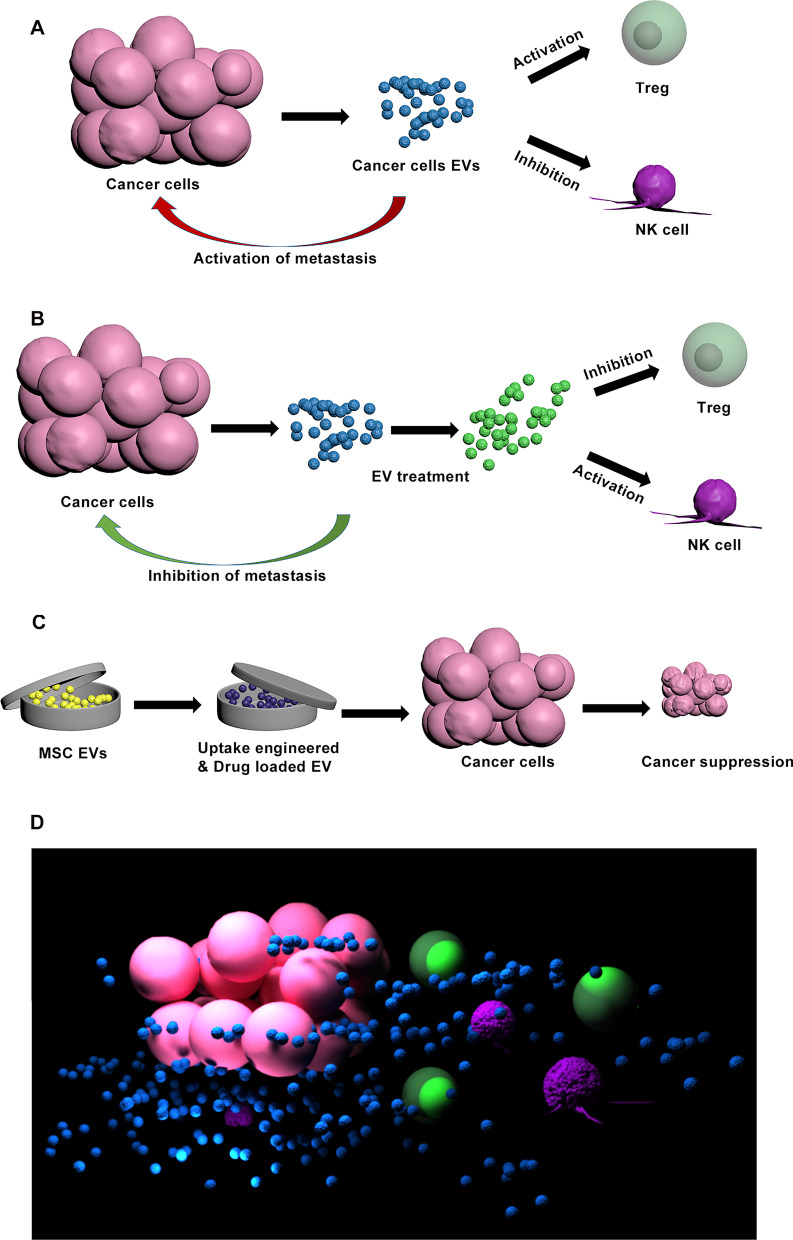
Schematic diagram that represents EV engineering for cancer therapy in clinical application. **A** Activation of immunomodulatory cells (including Treg) and inhibition of cytotoxic cells (including NK cells) by cancer cell EVs in order to suppress immune response and lead to metastasis. **B** Treatment of cancer cell EV to cease EV uptake by immune cells, in order to activate immune response and inhibit metastasis. **C** Loading chemotherapy drugs into EV and engineering EV uptake to treat cancers. **D** Three dimensional image of immunomodulatory cells (Green) and cytotoxic cells (Purple) which are respectively activated and inhibited by EVs (Blue) secreted by cancer cells (Pink)

For ideal EV therapy, selective or targeted EV uptake should be inhibited in the recipient cells. For example, selective prevention of EV (that is released from cancer cells) absorption by immune cells could prohibit their suppression and promote cancer treatment (Fig. 1). Therefore, finding a proper method for treatment of secreted EVs or immune cells could be a valuable asset for disease treatment.

##### Treatment of recipient cell extracellular matrix (ECM) components

EVs have a uniquely large surface area that can interact with both cells and biomolecules in the extracellular microenvironment [85]. The ECM components are effective in EV trapping and uptake. For example, heparan sulfate proteoglycans (HSPGs) are found at the cell surface and in the ECM. Various complexes, including viral particles and lipoproteins, use HSPGs to facilitate their transfer into cells [86]. Fibronectin is an ECM component that could simultaneously bind to HSPGs on the exosomal and plasma membrane surfaces, and facilitate cellular uptake of EVs [87]. A decrease in EV uptake following treatment with heparin sulfate mimetic molecules may be related to fibronectin binding [68]. Other ECM compounds may also play a role in EV uptake and blocking them can prevent this uptake. This field appears to have tremendous potential for research and clinical applications.

## Extracellular vesicle (EV) uptake customization by novel EV engineering strategies

Despite significant improvements in the traditional strategies for customizing EV uptake, there are numerous problems that exist such as rapid clearance, low EV uptake rate by recipient cells, and off-target effects. In recent years, research has shown that EV engineering can overcome these limitations; therefore, EV uptake can be customized as targeted drug delivery systems [43, 88, 89].

EV engineering procedures are performed by direct or indirect strategies in the presence and absence of genetic manipulation, which has been previously reviewed [7]. We intend to discuss recent advances in EV uptake engineering strategies in terms of customization and targeting of EV uptake.

### Extracellular vesicle (EV) engineering strategies for improvement of innate targeting capacity

EVs have innate targeting that can be upgraded by EV engineering strategies to increase the efficiency of EV therapy (Table 2). The results of numerous reports have shown the innate capacity of EVs for specific cells or tissues. For example, EVs secreted by cortical neurons were selectively absorbed by neurons [90], and MSC-exosomes specifically accumulated in the kidneys of a mouse model of glycerol-induced acute kidney injury compared to the healthy group [91]. Intrinsic tissue tropism and selective uptake of EVs depend on superficial and integral EV cargos [92]. EV tropism can be engineered directly or indirectly in order to specifically modify the innate targeting of recipient cells. The existence of different integrins, CD63, complex of the tetraspanin 8 (TSPAN8), integrin α4, and glycans on the surface of the EV determines its tropism [79, 83, 93]. All have the capability to be engineered and regulate EV uptake [81]. Internal cargos are involved in EV tropism. For example, circulating exosomal miRNAs play a role in organotropism of breast cancer metastasis [94]. Overexpression of Wnt4 in donor cells led to production of transgenic exosomes that showed increased homing to the thymus compared to the un-engineered exosomes.[95]. A negative selection mechanism, by overexpression of CD47 on the EV surface, prevented EVs from uptake and elimination by phagocytic cells [96, 97], which resulted in an increased chance of EV uptake by their targeted cells.

**Table 2:** Extracellular vesicle (EV) treatment for preclinical studies in disease models

Disease models	In vivo/in vitro	Administration route	Delivery (sustained release/injection)	Engineered/non-engineered	Methods for Enhancing EVs therapeutic effects	References
Chronic liver failure	In vivo	Systemic	Sustained release	Hydrogel-mediated	–	[62]
Alzheimer’s disease	In vivo	Systemic	Injection	Targeted	Engineering the dendritic cells to express Lamp2b fused to the neuron-specific RVG peptide for delivering exogenous siRNA	[43]
Breast cancer	In vitro	–	–	Engineered-targeted	HEK293T cells transduced by a lentiviral vector bearing-LAMP2b-DARPin G3 chimeric gene for siRNA delivering	[88]
Parkinson’s disease	In vivo/ in vitro	Systemic	–	Engineered	Catalase loading into exosomes by different methods	[125]
Cartilage damage	In vivo/in vitro	Local	Injection	–	–	[5]
Osteoarthritis	In vivo	Local	Injection	Engineered	miR-140-5p-overexpressing in human synovial MSCs for the production of enriched EV	[4]

The innate targeting of EV has high potential in cancer therapy. Exosomes secreted by metastatic cancer cells could be preferentially uptaken by specific host organ to organize the pre-metastatic niche via upregulation of proinflammatory gene expression and immunosuppressive cytokine, which leads to organotropic metastasis [98]. The amount and origin of EVs affect their organ-specific uptake during metastasis as the rate of EV uptake secreted by malignant cancer cells is more than benign cancer cells [99]. It has also been shown that targeting the integrins α6β4 and αvβ5 of exosomes reduced their uptake and metastasis to lung and liver, respectively [83]. Hence, metastasis might be controllable provided the recipient cells are prevented from uptaking these EVs, although this approach requires further research.

Since EVs are safe and have the capacity to carry desired antigens and deliver them to immune cells, they can be used for vaccine production. Dendritic cells as antigen-presenting cells regulate immune responses by releasing their exosomes that are innately uptake by immune cells such as T cells and B cells. EVs secreted by dendritic cells can be loaded by viral proteins (as a superficial or internal cargo) or mRNAs (as an internal cargo) in order to severely elevate specific CD8 ( +) T cell and B cell reactions and create more effective immunity [100]. Recently, Tsai et al. has developed a COVID-19 vaccine by the EV-based mRNA delivery for the expression of viral antigens. After uptake and cargo delivery of these EVs, antigen-presenting cells express several viral antigen proteins that evoke CD4 + and CD8 + T cells for effective immune responses [101]. These EVs could be engineered for the improvement of their targeted delivery by tetraspanins or other superficial proteins.

Overall, our knowledge about the innate organotropism of EVs is still in its infancy, and an accurate understanding of its mechanisms would be beneficial in the treatment of diseases. It seems that engineering of the EV surface and internal cargos have tremendous potential to improve innate tropism in targeted EV uptake by their natural recipient cells for EV therapy.

### Extracellular vesicle (EV) engineering strategies for artificial targeting

In this approach, we can customize EV uptake by increasing its affinity to the desired artificial recipient cells. For this purpose, researchers designed specific molecules that are synthetic mediators on the EV surface to specifically bind to a molecule at the membrane surface of the desired target cells. Thus far, a number of bioengineering strategies have been developed, which can be categorized into four discrete approaches: receptor-ligand, enzymatic, and antigen–antibody or their combination.

In terms of the receptor-ligand approach, researchers modified EVs with ligands that could specifically bind to targeted cells [102]. For example, EVs were bioengineered to specifically bond to HER2/Neu by expressing designed ankyrin repeat proteins (DARPins) on the cancer cell membrane surface [88]. In order to deliver small interfering RNAs (siRNA) specifically to brain cells, the EVs were isolated form dendritic cells that were genetically engineered to express Lamp2b, an exosomal membrane protein. Lamp2b fuses to the neuron-specific rabies viral glycoprotein (RVG) peptide. This approach led to an increase in targeted EV uptake by neurons [43]. Transferrin-conjugated magnetic particles bound to a transferrin receptor on the EVs surface increased EV uptake by cancer cells in the presence of an external magnetic field, and consequently suppressed tumor growth [103].

A second strategy has emerged that target antigens by specific antibodies. Antigens are biomolecules involved in ligand-receptor interactions that have the ability to stimulate the host immune response. Epidermal growth factor receptor (EGFR) is overexpressed in cancer cells. Therefore, Cheng et al. engineered anti-CD3 and anti-EGFR on the surfaces of exosomes to cross-link T cells and EGFR + cancer cells in order for the T cells to eliminate the cancer cells [89]. Recombinant fusion proteins, including nanobodies against the EGFR and lactadherin (C1C2) domains could bind to phosphatidylserine (PS) on the EV surface by C1C2. Therefore, this recombinant protein could provide a specific binding site and boost cancer cell uptake of the EVs that contained an anti-cancer drug [104]. The addition of nanobodies on the surface of EVs via glycosylphosphatidylinositol (GPI) changes EV cell targeting by greatly improving EV binding to cancer cells for chemotherapy drug delivery [105]. There is the possibility to directly embed a tissue-specific antibody or homing peptide on the EV surface in order to facilitate their uptake by target cells, including cardiac fibroblasts, myoblasts and ischemic myocardium [1]. In order to improve muscle function in a mouse model of muscular dystrophy, researchers attached peptide CP05 to CD63 on the EV surface to change EV homing and biodistribution, and increase delivery of a splice-correcting oligomer to muscle cells [106].

Artificial chimeric exosome is a new strategy that could be useful for anti-phagocytosis and targeted cancer therapy. These artificial exosomes are constructed by integrating cell membrane proteins from multiple cell types (red blood cells and cancer cells) into synthetic phospholipid bilayers. [107]. A biomimetic artificial strategy is exploited to prepare liposome-like nanovesicles that artificially have a variety of targets for protein/peptide ligands such as anti-HER2 affibody (a type of small protein engineered to an antibody mimetic) while containing chemotherapy drugs for enhanced targeted drug delivery [108].

The use of enzymes that degrade ECM on the EV surface may increase EV uptake. Hyaluronan is a glycosaminoglycan that can accumulate in the ECM of tumors. GPI-anchored PH20 hyaluronidase on an exosome surface degraded tumor ECM and enhanced both T cell and drug permeability in the tumor milieu in order to destroy the cancer cells [109].

Bioengineering of EV surface molecules, production of chimeric and biomimetic EVs and, particularly the potential of tetraspanins in an EV membrane, are proposed strategies that could be efficient for disease treatment. Notably, targeted artificial EVs appear to have a promising future for treatment due to their potential to fully customize surface design and internal cargos.

### Extracellular vesicle (EV) engineering strategies for an extended circulation time and decreased clearance

One of the major issues in EV administration is their short half-life in the circulation because of their rapid clearance. There are various strategies to increase both the EV half-life and their uptake. The addition of polyethylene glycol (PEG) to the EV surface increases circulation time, cell specificity, and reduces immunogenicity [110]. They prepared epidermal growth factor receptor nanobody-PEG-lipids and then mixed with EVs. Kamerkar et al. have reported that the presence of CD47 on EV surface inhibited EV uptake and clearance from the circulation by macrophages and monocytes which increased the chance of EV uptake by recipient cells [97]. Accordingly, amplification of CD47 expression on the surface of EV through engineering of donor cells may help increase EV circulation time.

Macrophages recognize the negative charge of PS, which leads to an increased clearance of IV injected EVs [2]. We assume that reduction of PS groups in the EV membrane or neutralizing the surface negative charge would result in a decrease of EV uptake by macrophages.

Research on nanoparticles has shown that particle size affects their clearance [3] because nanoparticles smaller than 100 nm are less prone to elimination by macrophages. EVs are nanoparticles that apparently obey this rule.

All in all, the concurrent use of two or more of the previously mentioned approaches such as PEG, CD47, PS, or EV size would increase the EV circulation time in the blood and delay their clearance. This would increase the chances of EV uptake by recipient cells. Further research in this context is very promising for the therapeutic applications of EVs.

### Post extracellular vesicle (EV) uptake engineering strategies

**Fig. 2:**
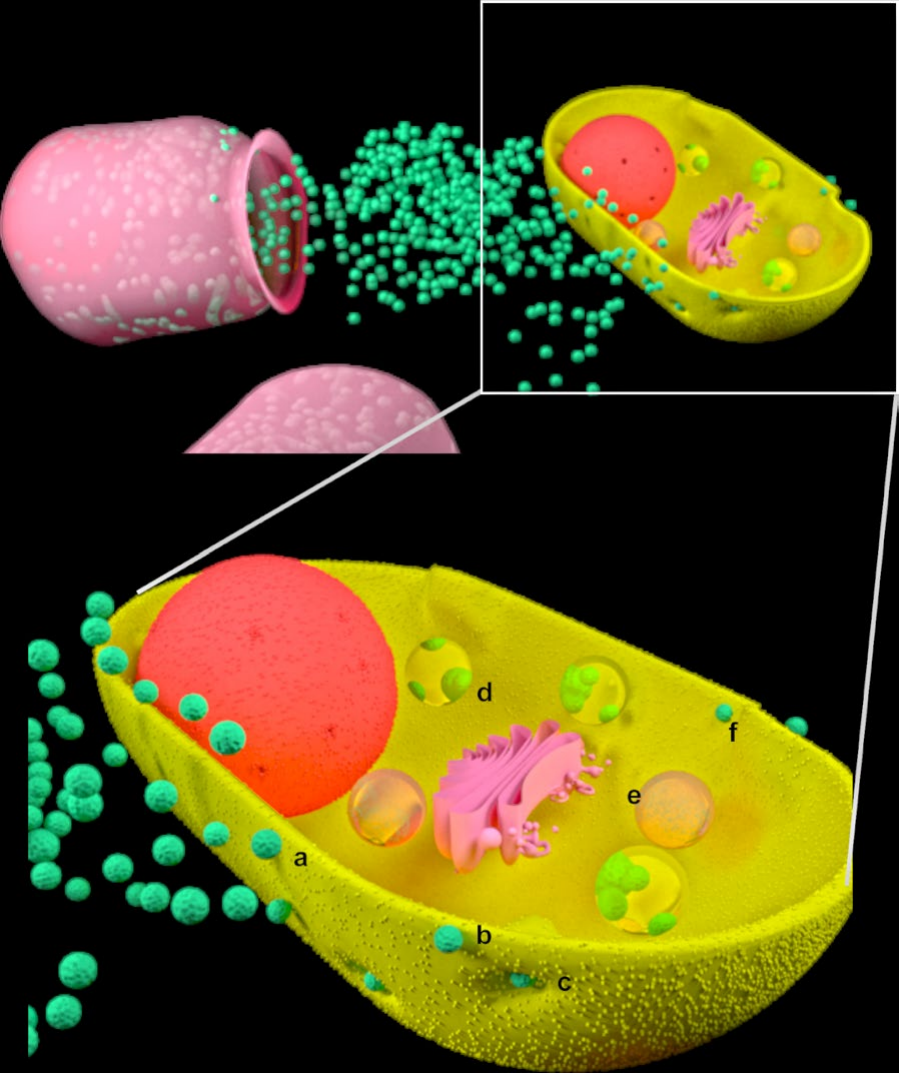
Schematic diagram that represents secretion of EVs by donor cells and EV uptake by recipient cell through contact without internalization (a), membrane fusion (b), internalization (c) and, post internalization fate of uptaken-EV including delivery (d), degradation (e) and recycling (f)

In order to have ideal use of EV capabilities for their uptake, the fate of absorbed EVs inside the recipient cell must be taken into consideration. After internalization, EVs undergo recycling, degradation, and delivery to the cytosol [111] or endoplasmic reticulum, depending on the type of disease and its progression [112–114] (Fig. 2, Table 3). EV should be engineered to increase their uptake and determine their fate in the receipt cells, which would likely result in a more efficient EV therapy. Nakase et al. designed a pH-sensitive fusion polypeptide and cationic lipid material to concurrently anchor on the surface of an exosome. Their findings showed improved cellular EV uptake and an efficient cytosolic release [111]. Conversely, neutralization of endosomal pH and cholesterol accumulation in endosomes by Bafilomycin A1, as a Vacuolar-type ATPase (V-ATPase) inhibitor, blocked cytosolic release of the endosomal cargos [114].

**Table 3:** Extracellular vesicle (EV) uptake routes

EV uptake route	Docking goal	Mechanism	Intracellular fate	References
Membrane fusion	Cargo release directly into the cytosol	Direct membrane fusion	Delivery of cargo directly into the cytosol	[96, 114]
Contact without internalization	Trigger signaling pathways	Signaling pathways	Activation of signaling pathways	[126]
Internalization	Internalization	Endocytosis	Recycling	[96, 114]
		Phagocytosis	Degradation	
		Macropinocytosis	Delivery	

EVs secreted from cancer cells are supposed to mediate cell–cell communication during metastasis [74], which is associated with an endosomal recycling pathway in cancer cells. Recycling endosomes is the re-release of internalized EVs to the extracellular space via recipient cells. For instance, recycling of internalized fibroblast-derived CD81 + EVs by breast cancer cells could trigger their migration for metastasis [115]. Therefore, we assume that metastasis could be inhibited through blocking EVs recycling and compel internalized EVs to be degraded.

Rab5 and Rab7 are small GTPases that regulate the essential steps in EV endocytosis, their cargo uptake into early endosomes, and transport to lysosomes for degradation [116]. Therefore, they may be good engineering candidates for post-EV uptake.

The best engineering approach should target several goals by using a limited number of modifications. Post-EV uptake engineering is still in its infancy, and its perspective research and clinical applications appear to be promising for EV therapy.

## Clinical translation of engineered EV

Preclinical studies have shown that the EV engineering strategies have a high potential for control of challenges allocated to EV administration and their subsequent clinical translation in an efficient way. Despite satisfaction with the benefits of engineered EV, their probable side effects should not be neglected. These side effects might be related to the process of preparing, engineering, isolating, purification, and administration routes of EV. Thus far, approximately 50 clinical trials related to EV have been recorded on clinicaltrials.gov, and some of them are designed based on internal cargo engineered EV (including exosomes loaded with curcumin, antigen, and siRNA against KrasG12D) [117]. It is predictable that the designing of clinical trials will soon reach the field of targeted EV uptake engineering.

Currently, the various methods for production, engineering, and applications, are used for EVs, it is expected that these different methods will gradually be standardized and defined. In this regard, Minimal Information for Studies of EVs (MISEV) guidelines that were released by the International Society for EVs (ISEV) are the important step for the standardization of research and clinical applications of EV. It seems due to more complex procedures, we need to develop special comprehensive supplement guidelines and standardization protocols of EV engineering for clinical application.

## Conclusion and future perspectives

**Fig. 3:**
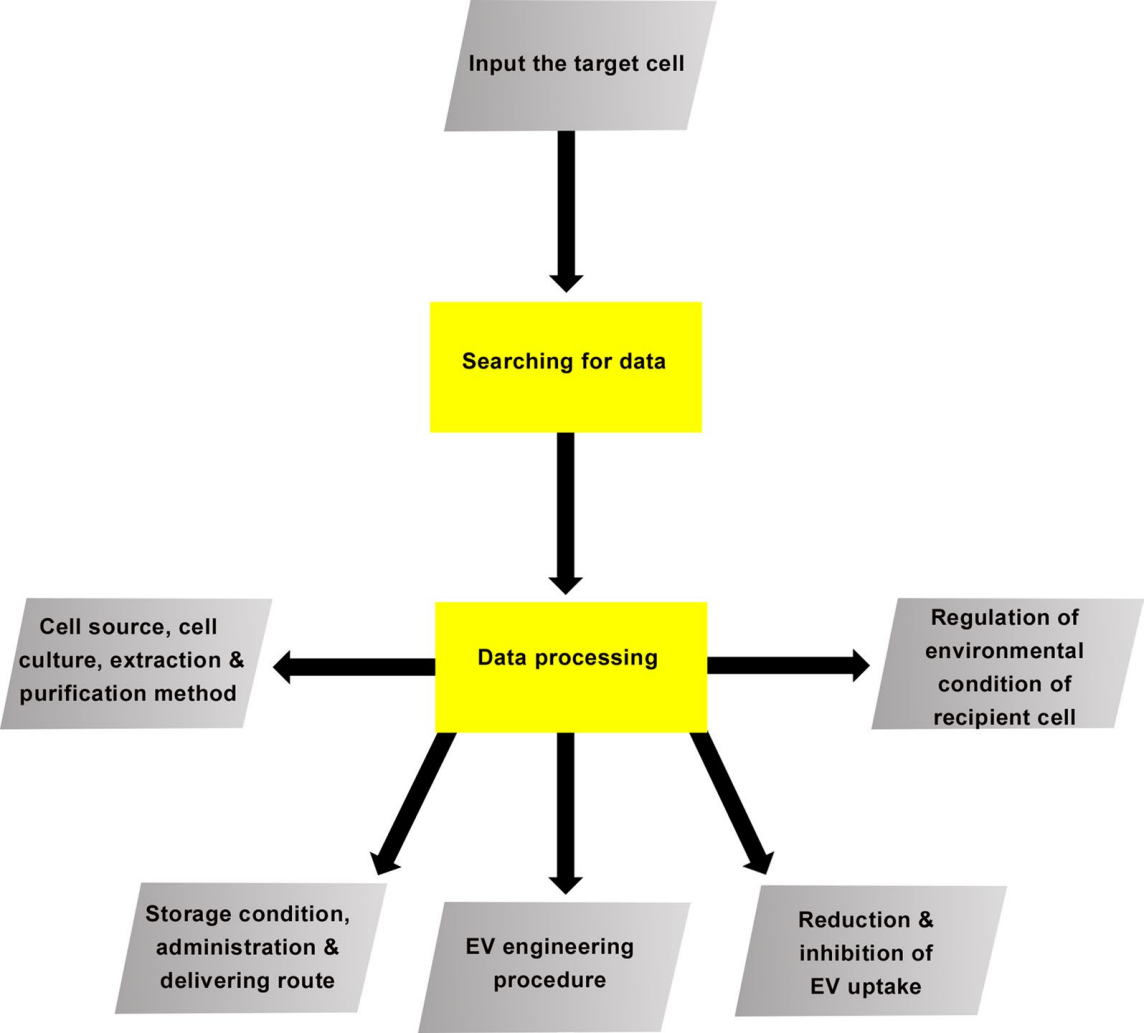
Schematic diagram that represents the overview of an algorithm that could be used to refer to relevant databases that receive the target cell and tissue names, obtaining the relevant recorded information from these tissues and, after processing, can provide output to researchers. The output could provide the best suggestions for: selecting a proper cell source, extracellular vesicle (EV) administration and delivery, and EV engineering and donor cells for customizing EV uptake for therapeutic use

In recent years, researchers have paid increasing attention to the therapeutic effects of EVs because of their innate therapeutic properties and capability to be engineered. In order to optimize the use of EV therapeutic properties, engineering methods should be developed to overcome the limitations and challenges that include rapid clearance of EVs and their targeted delivery. EVs have the potential to be engineered in terms of internal and superficial cargo for EV uptake. Therefore, although EVs have innate tropism, EV engineering could enhance their innate targeting of recipient cells and they can also be engineered artificially for the desired target cells. Hence, we could regulate and customize the EV uptake by the recipient cells and consequently upgrade the EV therapeutic efficiency. It seems that due to increasing progress in EV engineering, the future perspective of EV uptake engineering as disease treatment could be very promising, especially when using a combined strategy of traditional and engineering approaches that complement each other. Today, tremendous research has been conducted in terms of EV uptake. The increased amount of EV data mandates that researchers generate more comprehensive databases that can provide relevant services. One of the main services of these databases could pertain to customizing EVs absorption for research and clinic use because of the influence of numerous biological, biochemical, and biophysical factors. The best EV uptake engineering needs a professional, strong algorithm that asks the target cell and subsequently refers to the databases. According to available records, the most appropriate options for the best selection of the cell source, type of cell culture, extraction method, purification route, storage condition, administration route and delivery of the EVs to the recipient cells, and the method of treating the recipient cell and the best engineering method for donor cells and EVs could be determined to customize EV uptake. Figure 3 provides an outline of a required algorithm. Overall, increased research in EV uptake engineering for EV therapy appears promising and can make a considerable contribution to disease treatment in the near future.

## Data Availability

Not applicable.

## Abbreviations

DARPins: Designed ankyrin repeat proteins
ECM: Extracellular matrix
EGFR: Epidermal growth factor receptor
EV: Extracellular vesicles
GIP: Glycosylphosphatidylinositol
HSPGs: Heparan sulfate proteoglycans
IN: Intranasal
IP: Intraperitoneal
ITGB3: Integrin beta 3
IV: Intravenous
miRNA: MicroRNA
MSC: Mesenchymal stem cell
PEG: Poly ethylene glycol
PS: Phosphatidylserine
RVG: Rabies viral glycoprotein
SC: Subcutaneous
SEC: Size-exclusion chromatography
siRNAs: Small interfering RNAs
TSPAN8: Tetraspanin 8
UC: Umbilical cord
V-ATPase: Vacuolar-type ATPase

## References

[1] Antes TJ, Middleton RC, Luther KM, Ijichi T, Peck KA, Liu WJ, Valle J, Echavez AK, Marbán E (2018). Targeting extracellular vesicles to injured tissue using membrane cloaking and surface display. J Nanobiotechnol.

[2] Matsumoto A, Takahashi Y, Nishikawa M, Sano K, Morishita M, Charoenviriyakul C, Saji H, Takakura Y (2017). Role of phosphatidylserine-derived negative surface charges in the recognition and uptake of intravenously injected B16BL6-derived exosomes by macrophages. J Pharm Sci.

[3] Wiklander OPB, Nordin JZ, O'Loughlin A, Gustafsson Y, Corso G, Mäger I, Vader P, Lee Y, Sork H, Seow Y, Heldring N, Alvarez-Erviti L, Smith CIE, Le Blanc K, Macchiarini P, Jungebluth P, Wood MJA, Andaloussi SE (2015). Extracellular vesicle in vivo biodistribution is determined by cell source, route of administration and targeting. J Extracell Vesicles.

[4] Tao SC, Yuan T, Zhang YL, Yin WJ, Guo SC, Zhang CQ (2017). Exosomes derived from miR-140-5p-overexpressing human synovial mesenchymal stem cells enhance cartilage tissue regeneration and prevent osteoarthritis of the knee in a rat model. Theranostics.

[5] Headland SE, Jones HR, Norling LV, Kim A, Souza PR, Corsiero E, Gil CD, Nerviani A, Dell'Accio F, Pitzalis C, Oliani SM, Jan LY, Perretti M (2015). Neutrophil-derived microvesicles enter cartilage and protect the joint in inflammatory arthritis. Sci Transl Med.

[6] Zhao G, Liu F, Liu Z, Zuo K, Wang B, Zhang Y, Han X, Lian A, Wang Y, Liu M, Zou F, Li P, Liu X, Jin M, Liu JY (2020). MSC-derived exosomes attenuate cell death through suppressing AIF nucleus translocation and enhance cutaneous wound healing. Stem Cell Res Ther.

[7] Esmaeili A, Hosseini S, Baghaban Eslaminejad M (2021). Engineered-extracellular vesicles as an optimistic tool for microRNA delivery for osteoarthritis treatment. Cell Mol Life Sci.

[8] Apodaca LA, Baddour AAD, Garcia C, Alikhani L, Giedzinski E, Ru N, Agrawal A, Acharya MM, Baulch JE (2021). Human neural stem cell-derived extracellular vesicles mitigate hallmarks of Alzheimer’s disease. Alzheimer's Res Ther.

[9] Lai CP, Kim EY, Badr CE, Weissleder R, Mempel TR, Tannous BA, Breakefield XO (2015). Visualization and tracking of tumour extracellular vesicle delivery and RNA translation using multiplexed reporters. Nat Commun.

[10] Lázaro-Ibáñez E, Lunavat TR, Jang SC, Escobedo-Lucea C, Oliver-De La Cruz J, Siljander P, Lötvall J, Yliperttula M (2017). Distinct prostate cancer-related mRNA cargo in extracellular vesicle subsets from prostate cell lines. BMC Cancer.

[11] Zheng M, Huang M, Ma X, Chen H, Gao X (2019). Harnessing exosomes for the development of brain drug delivery systems. Bioconjug Chem.

[12] Chiou NT, Kageyama R, Ansel KM (2018). Selective export into extracellular vesicles and function of tRNA fragments during T cell activation. Cell Rep.

[13] Tran N (2016). Cancer exosomes as miRNA factories. Trends Cancer.

[14] Fitzgerald W, Freeman ML, Lederman MM, Vasilieva E, Romero R, Margolis L (2018). A system of cytokines encapsulated in extracellular vesicles. Sci Rep.

[15] Bian L, Zhai DY, Mauck RL, Burdick JA (2011). Coculture of human mesenchymal stem cells and articular chondrocytes reduces hypertrophy and enhances functional properties of engineered cartilage. Tissue Eng Part A.

[16] Kim M, Steinberg DR, Burdick JA, Mauck RL (2019). Extracellular vesicles mediate improved functional outcomes in engineered cartilage produced from MSC/chondrocyte cocultures. Proc Natl Acad Sci.

[17] Fukuta T, Nishikawa A, Kogure K (2020). Low level electricity increases the secretion of extracellular vesicles from cultured cells. Biochem Biophys Rep.

[18] Glebov K, Löchner M, Jabs R, Lau T, Merkel O, Schloss P, Steinhäuser C, Walter J (2015). Serotonin stimulates secretion of exosomes from microglia cells. Glia.

[19] Segura E, Nicco C, Lombard B, Véron P, Raposo G, Batteux F, Amigorena S, Théry C (2005). ICAM-1 on exosomes from mature dendritic cells is critical for efficient naive T-cell priming. Blood.

[20] Boussadia Z, Lamberti J, Mattei F, Pizzi E, Puglisi R, Zanetti C, Pasquini L, Fratini F, Fantozzi L, Felicetti F, Fecchi K, Raggi C, Sanchez M, D'Atri S, Carè A, Sargiacomo M, Parolini I (2018). Acidic microenvironment plays a key role in human melanoma progression through a sustained exosome mediated transfer of clinically relevant metastatic molecules. J Exp Clin Cancer Res CR.

[21] Cha JM, Shin EK, Sung JH, Moon GJ, Kim EH, Cho YH, Park HD, Bae H, Kim J, Bang OY (2018). Efficient scalable production of therapeutic microvesicles derived from human mesenchymal stem cells. Sci Rep.

[22] Rocha S, Carvalho J, Oliveira P, Voglstaetter M, Schvartz D, Thomsen AR, Walter N, Khanduri R, Sanchez J-C, Keller A, Oliveira C, Nazarenko I (2019). 3D cellular architecture affects MicroRNA and protein cargo of extracellular vesicles. Adv Sci.

[23] Zhang Y, Chopp M, Zhang ZG, Katakowski M, Xin H, Qu C, Ali M, Mahmood A, Xiong Y (2017). Systemic administration of cell-free exosomes generated by human bone marrow derived mesenchymal stem cells cultured under 2D and 3D conditions improves functional recovery in rats after traumatic brain injury. Neurochem Int.

[24] Haraszti RA, Miller R, Stoppato M, Sere YY, Coles A, Didiot MC, Wollacott R, Sapp E, Dubuke ML, Li X, Shaffer SA, DiFiglia M, Wang Y, Aronin N, Khvorova A (2018). Exosomes produced from 3D cultures of MSCs by tangential flow filtration show higher yield and improved activity. Mol Ther.

[25] Caponnetto F, Manini I, Skrap M, Palmai-Pallag T, di Loreto C, Beltrami A, Cesselli D, Ferrari E (2016). Size-dependent cellular uptake of exosomes. Nanomed Nanotechnol Biol Med.

[26] Yang Y, Tai X, Shi K, Ruan S, Qiu Y, Zhang Z, Xiang B, He Q (2016). A New concept of enhancing immuno-chemotherapeutic effects against B16F10 tumor via systemic administration by taking advantages of the limitation of EPR effect. Theranostics.

[27] Danaei M, Dehghankhold M, Ataei S, Hasanzadeh Davarani F, Javanmard R, Dokhani A, Khorasani S, Mozafari MR (2018). Impact of particle size and polydispersity index on the clinical applications of lipidic nanocarrier systems. Pharmaceutics.

[28] Zwi-Dantsis L, Winter CW, Kauscher U, Ferrini A, Wang B, Whittaker TE, Hood SR, Terracciano CM, Stevens MM (2020). Highly purified extracellular vesicles from human cardiomyocytes demonstrate preferential uptake by human endothelial cells. Nanoscale.

[29] Tian Y, Gong M, Hu Y, Liu H, Zhang W, Zhang M, Hu X, Aubert D, Zhu S, Wu L, Yan X (2020). Quality and efficiency assessment of six extracellular vesicle isolation methods by nano-flow cytometry. J Extracell Vesicles.

[30] McNamara RP, Dittmer DP (2020). Modern techniques for the isolation of extracellular vesicles and viruses. J Neuroimmune Pharmacol.

[31] Brown PN, Yin H (2017). Polymer-based purification of extracellular vesicles. Methods Mol Biol.

[32] Rider MA, Hurwitz SN, Meckes DG (2016). ExtraPEG: a polyethylene glycol-based method for enrichment of extracellular vesicles. Sci Rep.

[33] Balaj L, Atai NA, Chen W, Mu D, Tannous BA, Breakefield XO, Skog J, Maguire CA (2015). Heparin affinity purification of extracellular vesicles. Sci Rep.

[34] Maroto R, Zhao Y, Jamaluddin M, Popov VL, Wang H, Kalubowilage M, Zhang Y, Luisi J, Sun H, Culbertson CT, Bossmann SH, Motamedi M, Brasier AR (2017). Effects of storage temperature on airway exosome integrity for diagnostic and functional analyses. J Extracell Vesicles.

[35] Park S, Jeon H, Yoo S-M, Lee M-S (2018). The effect of storage temperature on the biological activity of extracellular vesicles for the complement system. In Vitro Cell Dev Biol Anim.

[36] Lee M, Ban J-J, Im W, Kim M (2016). Influence of storage condition on exosome recovery. Biotechnol Bioprocess Eng.

[37] Cheng Y, Zeng Q, Han Q, Xia W (2018). Effect of pH, temperature and freezing-thawing on quantity changes and cellular uptake of exosomes. Protein Cell.

[38] Mastazliha TS, Shima W, Berahim Z (2018). Establishment of the collection, Storage and preservation methods and their influence on stability of human salivary exosome. J Biomed Clin Sci.

[39] Bosch S, de Beaurepaire L, Allard M, Mosser M, Heichette C, Chrétien D, Jegou D, Bach J-M (2016). Trehalose prevents aggregation of exosomes and cryodamage. Sci Rep.

[40] Kusuma GD, Barabadi M, Tan JL, Morton DAV, Frith JE, Lim R (2018). To protect and to preserve: novel preservation strategies for extracellular vesicles. Front Pharmacol.

[41] Ban JJ, Lee M, Im W, Kim M (2015). Low pH increases the yield of exosome isolation. Biochem Biophys Res Commun.

[42] Sokolova V, Ludwig A-K, Hornung S, Rotan O, Horn PA, Epple M, Giebel B (2011). Characterisation of exosomes derived from human cells by nanoparticle tracking analysis and scanning electron microscopy. Colloids Surf B.

[43] Alvarez-Erviti L, Seow Y, Yin H, Betts C, Lakhal S, Wood MJ (2011). Delivery of siRNA to the mouse brain by systemic injection of targeted exosomes. Nat Biotechnol.

[44] Lun XQ, Jang JH, Tang N, Deng H, Head R, Bell JC, Stojdl DF, Nutt CL, Senger DL, Forsyth PA, McCart JA (2009). Efficacy of systemically administered oncolytic vaccinia virotherapy for malignant gliomas is enhanced by combination therapy with rapamycin or cyclophosphamide. Clin Cancer Res Off J Am Assoc Cancer Res.

[45] Imai T, Takahashi Y, Nishikawa M, Kato K, Morishita M, Yamashita T, Matsumoto A, Charoenviriyakul C, Takakura Y (2015). Macrophage-dependent clearance of systemically administered B16BL6-derived exosomes from the blood circulation in mice. J Extracell Vesicles.

[46] Takahashi Y, Nishikawa M, Shinotsuka H, Matsui Y, Ohara S, Imai T, Takakura Y (2013). Visualization and in vivo tracking of the exosomes of murine melanoma B16-BL6 cells in mice after intravenous injection. J Biotechnol.

[47] Scrimgeour LA, Potz BA, Aboul Gheit A, Liu Y, Shi G, Pfeiffer M, Colantuono BJ, Sodha NR, Abid MR, Sellke FW (2020). Intravenous injection of extracellular vesicles to treat chronic myocardial ischemia. PLoS ONE.

[48] Milano G, Biemmi V, Lazzarini E, Balbi C, Ciullo A, Bolis S, Ameri P, Di Silvestre D, Mauri P, Barile L, Vassalli G (2019). Intravenous administration of cardiac progenitor cell-derived exosomes protects against doxorubicin/trastuzumab-induced cardiac toxicity. Cardiovasc Res.

[49] Lai CP, Mardini O, Ericsson M, Prabhakar S, Maguire C, Chen JW, Tannous BA, Breakefield XO (2014). Dynamic biodistribution of extracellular vesicles in vivo using a multimodal imaging reporter. ACS Nano.

[50] Shin K-O, Ha DH, Kim JO, Crumrine DA, Meyer JM, Wakefield JS, Lee Y, Kim B, Kim S, Kim H-K, Lee J, Kwon HH, Park G-H, Lee JH, Lim J, Park S, Elias PM, Park K, Yi YW, Cho BS (2020). Exosomes from human adipose tissue-derived mesenchymal stem cells promote epidermal barrier repair by inducing de novo synthesis of ceramides in atopic dermatitis. Cells.

[51] Lu M, Peng L, Ming X, Wang X, Cui A, Li Y, Wang X, Meng D, Sun N, Xiang M, Chen S (2019). Enhanced wound healing promotion by immune response-free monkey autologous iPSCs and exosomes vs. their allogeneic counterparts. EBioMedicine.

[52] Yang J, Chen Z, Pan D, Li H, Shen J (2020). Umbilical cord-derived mesenchymal stem cell-derived exosomes combined pluronic F127 hydrogel promote chronic diabetic wound healing and complete skin regeneration. Int J Nanomed.

[53] Kodali M, Castro OW, Kim D-K, Thomas A, Shuai B, Attaluri S, Upadhya R, Gitai D, Madhu LN, Prockop DJ, Shetty AK (2019). Intranasally administered human msc-derived extracellular vesicles pervasively incorporate into neurons and microglia in both intact and status epilepticus injured forebrain. Int J Mol Sci.

[54] Long Q, Upadhya D, Hattiangady B, Kim DK, An SY, Shuai B, Prockop DJ, Shetty AK (2017). Intranasal MSC-derived A1-exosomes ease inflammation, and prevent abnormal neurogenesis and memory dysfunction after status epilepticus. Proc Natl Acad Sci USA.

[55] Narbute K, Piļipenko V, Pupure J, Dzirkale Z, Jonavičė U, Tunaitis V, Kriaučiūnaitė K, Jarmalavičiūtė A, Jansone B, Kluša V, Pivoriūnas A (2019). Intranasal administration of extracellular vesicles derived from human teeth stem cells improves motor symptoms and normalizes tyrosine hydroxylase expression in the substantia Nigra and striatum of the 6-hydroxydopamine-treated rats. Stem Cells Transl Med.

[56] ClinicalTrials.gov. A pilot clinical study on inhalation of mesenchymal stem cells exosomes treating severe novel coronavirus pneumonia [Internet], Bethesda: 2020. Registration number: NCT04276987.

[57] ClinicalTrials.gov. A tolerance clinical study on aerosol inhalation of mesenchymal stem cells exosomes in healthy volunteers [Internet], Bethesda: 2020. Registration number: NCT04313647.

[58] Chinese Clinical Trial Register. A study for the key technology of mesenchymal stem cells exosomes atomization in the treatment of novel coronavirus pneumonia (COVID-19) [Internet], Chengdu: Ministry of Health; 2020. Registration number: ChiCTR2000030261.

[59] Dinh P-UC, Paudel D, Brochu H, Popowski KD, Gracieux MC, Cores J, Huang K, Hensley MT, Harrell E, Vandergriff AC, George AK, Barrio RT, Hu S, Allen TA, Blackburn K, Caranasos TG, Peng X, Schnabel LV, Adler KB, Lobo LJ, Goshe MB, Cheng K (2020). Inhalation of lung spheroid cell secretome and exosomes promotes lung repair in pulmonary fibrosis. Nat Commun.

[60] Zhang S, Chu WC, Lai RC, Lim SK, Hui JH, Toh WS (2016). Exosomes derived from human embryonic mesenchymal stem cells promote osteochondral regeneration. Osteoarthr Cartil.

[61] Rohde E, Pachler K, Gimona M (2019). Manufacturing and characterization of extracellular vesicles from umbilical cord-derived mesenchymal stromal cells for clinical testing. Cytotherapy.

[62] Mardpour S, Ghanian MH, Sadeghi-Abandansari H, Mardpour S, Nazari A, Shekari F, Baharvand H (2019). Hydrogel-mediated sustained systemic delivery of mesenchymal stem cell-derived extracellular vesicles improves hepatic regeneration in chronic liver failure. ACS Appl Mater Interfaces.

[63] Han C, Zhou J, Liang C, Liu B, Pan X, Zhang Y, Wang Y, Yan B, Xie W, Liu F, Yu XY, Li Y (2019). Human umbilical cord mesenchymal stem cell derived exosomes encapsulated in functional peptide hydrogels promote cardiac repair. Biomater Sci.

[64] Abello J, Nguyen TDT, Marasini R, Aryal S, Weiss ML (2019). Biodistribution of gadolinium- and near infrared-labeled human umbilical cord mesenchymal stromal cell-derived exosomes in tumor bearing mice. Theranostics.

[65] Busatto S, Giacomini A, Montis C, Ronca R, Bergese P (2018). Uptake profiles of human serum exosomes by murine and human tumor cells through combined use of colloidal nanoplasmonics and flow cytofluorimetric analysis. Anal Chem.

[66] Costa Verdera H, Gitz-Francois JJ, Schiffelers RM, Vader P (2017). Cellular uptake of extracellular vesicles is mediated by clathrin-independent endocytosis and micropinocytosis. J Control Release Off J Control Release Soc.

[67] Reclusa P, Verstraelen P, Taverna S, Gunasekaran M, Pucci M, Pintelon I, Claes N, de Miguel-Pérez D, Alessandro R, Bals S, Kaushal S, Rolfo C (2020). Improving extracellular vesicles visualization: From static to motion. Sci Rep.

[68] Franzen CA, Simms PE, Van Huis AF, Foreman KE, Kuo PC, Gupta GN (2014). Characterization of uptake and internalization of exosomes by bladder cancer cells. Biomed Res Int.

[69] Jurgielewicz BJ, Yao Y, Stice SL (2020). Kinetics and specificity of HEK293T extracellular vesicle uptake using imaging flow cytometry. Nanoscale Res Lett.

[70] Svensson KJ, Christianson HC, Wittrup A, Bourseau-Guilmain E, Lindqvist E, Svensson LM, Mörgelin M, Belting M (2013). Exosome uptake depends on ERK1/2-heat shock protein 27 signaling and lipid Raft-mediated endocytosis negatively regulated by caveolin-1. J Biol Chem.

[71] Harrison SC (2008). Viral membrane fusion. Nat Struct Mol Biol.

[72] Mulcahy LA, Pink RC, Carter DR (2014). Routes and mechanisms of extracellular vesicle uptake. J Extracell Vesicles.

[73] Tian T, Zhu YL, Hu FH, Wang YY, Huang NP, Xiao ZD (2013). Dynamics of exosome internalization and trafficking. J Cell Physiol.

[74] Becker A, Thakur BK, Weiss JM, Kim HS, Peinado H, Lyden D (2016). Extracellular vesicles in cancer: cell-to-cell mediators of metastasis. Cancer Cell.

[75] Khan FM, Saleh E, Alawadhi H, Harati R, Zimmermann W-H, El-Awady R (2018). Inhibition of exosome release by ketotifen enhances sensitivity of cancer cells to doxorubicin. Cancer Biol Ther.

[76] Choi HI, Choi JP, Seo J, Kim BJ, Rho M, Han JK, Kim JG (2017). Helicobacter pylori-derived extracellular vesicles increased in the gastric juices of gastric adenocarcinoma patients and induced inflammation mainly via specific targeting of gastric epithelial cells. Exp Mol Med.

[77] Nishida-Aoki N, Tominaga N, Takeshita F, Sonoda H, Yoshioka Y, Ochiya T (2017). Disruption of circulating extracellular vesicles as a novel therapeutic strategy against cancer metastasis. Mol Ther.

[78] Nazarenko I, Rana S, Baumann A, McAlear J, Hellwig A, Trendelenburg M, Lochnit G, Preissner KT, Zöller M (2010). Cell surface tetraspanin Tspan8 contributes to molecular pathways of exosome-induced endothelial cell activation. Can Res.

[79] Rana S, Yue S, Stadel D, Zöller M (2012). Toward tailored exosomes: the exosomal tetraspanin web contributes to target cell selection. Int J Biochem Cell Biol.

[80] Morelli AE, Larregina AT, Shufesky WJ, Sullivan ML, Stolz DB, Papworth GD, Zahorchak AF, Logar AJ, Wang Z, Watkins SC, Falo LD (2004). Endocytosis, intracellular sorting, and processing of exosomes by dendritic cells. Blood.

[81] Williams C, Pazos R, Royo F, González E, Roura-Ferrer M, Martinez A, Gamiz J, Reichardt N-C, Falcón-Pérez JM (2019). Assessing the role of surface glycans of extracellular vesicles on cellular uptake. Sci Rep.

[82] Fuentes P, Sesé M, Guijarro PJ, Emperador M, Sánchez-Redondo S, Peinado H, Hümmer S, Ramón y Cajal S (2020). ITGB3-mediated uptake of small extracellular vesicles facilitates intercellular communication in breast cancer cells. Nat Commun.

[83] Hoshino A, Costa-Silva B, Shen TL, Rodrigues G, Hashimoto A, Tesic Mark M, Molina H, Kohsaka S, Di Giannatale A, Ceder S, Singh S, Williams C, Soplop N, Uryu K, Pharmer L, King T, Bojmar L, Davies AE, Ararso Y, Zhang T, Zhang H, Hernandez J, Weiss JM, Dumont-Cole VD, Kramer K, Wexler LH, Narendran A, Schwartz GK, Healey JH, Sandstrom P, Labori KJ, Kure EH, Grandgenett PM, Hollingsworth MA, de Sousa M, Kaur S, Jain M, Mallya K, Batra SK, Jarnagin WR, Brady MS, Fodstad O, Muller V, Pantel K, Minn AJ, Bissell MJ, Garcia BA, Kang Y, Rajasekhar VK, Ghajar CM, Matei I, Peinado H, Bromberg J, Lyden D (2015). Tumour exosome integrins determine organotropic metastasis. Nature.

[84] Fitzner D, Schnaars M, van Rossum D, Krishnamoorthy G, Dibaj P, Bakhti M, Regen T, Hanisch UK, Simons M (2011). Selective transfer of exosomes from oligodendrocytes to microglia by macropinocytosis. J Cell Sci.

[85] Buzás EI, Tóth EÁ, Sódar BW, Szabó-Taylor KÉ (2018). Molecular interactions at the surface of extracellular vesicles. Semin Immunopathol.

[86] Shukla D, Liu J, Blaiklock P, Shworak NW, Bai X, Esko JD, Cohen GH, Eisenberg RJ, Rosenberg RD, Spear PG (1999). A novel role for 3-O-sulfated heparan sulfate in herpes simplex virus 1 entry. Cell.

[87] Purushothaman A, Bandari SK, Liu J, Mobley JA, Brown EE, Sanderson RD (2015). Fibronectin on the surface of myeloma cell-derived exosomes mediates exosome-cell interactions. J Biol Chem.

[88] Limoni SK, Moghadam MF, Moazzeni SM, Gomari H, Salimi F (2019). Engineered exosomes for targeted transfer of siRNA to HER2 positive breast cancer cells. Appl Biochem Biotechnol.

[89] Cheng Q, Shi X, Han M, Smbatyan G, Lenz H-J, Zhang Y (2018). Reprogramming exosomes as nanoscale controllers of cellular immunity. J Am Chem Soc.

[90] Chivet M, Javalet C, Laulagnier K, Blot B, Hemming FJ, Sadoul R (2014). Exosomes secreted by cortical neurons upon glutamatergic synapse activation specifically interact with neurons. J Extracell Vesicles.

[91] Grange C, Tapparo M, Bruno S, Chatterjee D, Quesenberry PJ, Tetta C, Camussi G (2014). Biodistribution of mesenchymal stem cell-derived extracellular vesicles in a model of acute kidney injury monitored by optical imaging. Int J Mol Med.

[92] Murphy DE, de Jong OG, Brouwer M, Wood MJ, Lavieu G, Schiffelers RM, Vader P (2019). Extracellular vesicle-based therapeutics: natural versus engineered targeting and trafficking. Exp Mol Med.

[93] Laulagnier K, Javalet C, Hemming FJ, Chivet M, Lachenal G, Blot B, Chatellard C, Sadoul R (2018). Amyloid precursor protein products concentrate in a subset of exosomes specifically endocytosed by neurons. Cell Mol Life Sci CMLS.

[94] Wong GL, Abu Jalboush S, Lo H-W (2020). Exosomal MicroRNAs and organotropism in breast cancer metastasis. Cancers.

[95] Banfai K, Garai K, Ernszt D, Pongracz JE, Kvell K (2019). Transgenic exosomes for thymus regeneration. Front Immunol.

[96] Mathieu M, Martin-Jaular L, Lavieu G, Théry C (2019). Specificities of secretion and uptake of exosomes and other extracellular vesicles for cell-to-cell communication. Nat Cell Biol.

[97] Kamerkar S, LeBleu VS, Sugimoto H, Yang S, Ruivo CF, Melo SA, Lee JJ, Kalluri R (2017). Exosomes facilitate therapeutic targeting of oncogenic KRAS in pancreatic cancer. Nature.

[98] Chen W, Hoffmann AD, Liu H, Liu X (2018). Organotropism: new insights into molecular mechanisms of breast cancer metastasis. npj Precision Oncology.

[99] Linxweiler J, Kolbinger A, Himbert D, Zeuschner P, Saar M, Stöckle M, Junker K (2021). Organ-specific uptake of extracellular vesicles secreted by urological cancer cells. Cancers.

[100] Näslund TI, Gehrmann U, Qazi KR, Karlsson MC, Gabrielsson S (2013). Dendritic cell-derived exosomes need to activate both T and B cells to induce antitumor immunity. J Immunol.

[101] S.-J. Tsai, C. Guo, N.A. Atai, S.J. Gould, Exosome-mediated mRNA delivery for SARS-CoV-2 vaccination, bioRxiv (2020) 2020.11.06.371419. 10.1101/2020.11.06.371419.

[102] Meng W, He C, Hao Y, Wang L, Li L, Zhu G (2020). Prospects and challenges of extracellular vesicle-based drug delivery system: considering cell source. Drug Deliv.

[103] Qi H, Liu C, Long L, Ren Y, Zhang S, Chang X, Qian X, Jia H, Zhao J, Sun J, Hou X, Yuan X, Kang C (2016). Blood exosomes endowed with magnetic and targeting properties for cancer therapy. ACS Nano.

[104] Kooijmans SAA, Gitz-Francois J, Schiffelers RM, Vader P (2018). Recombinant phosphatidylserine-binding nanobodies for targeting of extracellular vesicles to tumor cells: a plug-and-play approach. Nanoscale.

[105] Kooijmans SA, Aleza CG, Roffler SR, van Solinge WW, Vader P, Schiffelers RM (2016). Display of GPI-anchored anti-EGFR nanobodies on extracellular vesicles promotes tumour cell targeting. J Extracell Vesicles.

[106] Gao X, Ran N, Dong X, Zuo B, Yang R, Zhou Q, Moulton HM, Seow Y, Yin H (2018). Anchor peptide captures, targets, and loads exosomes of diverse origins for diagnostics and therapy. Sci Transl Med.

[107] Zhang K-L, Wang Y-J, Sun J, Zhou J, Xing C, Huang G, Li J, Yang H (2018). Artificial chimeric exosomes for anti-phagocytosis and targeted cancer therapy. Chem Sci.

[108] Zhang P, Zhang L, Qin Z, Hua S, Guo Z, Chu C, Lin H, Zhang Y, Li W, Zhang X, Chen X, Liu G (2018). Genetically engineered liposome-like nanovesicles as active targeted transport platform. Adv Mater.

[109] Hong Y, Nam G-H, Koh E, Jeon S, Kim GB, Jeong C, Kim D-H, Yang Y, Kim I-S (2018). Exosome as a vehicle for delivery of membrane protein therapeutics, PH20, for enhanced tumor penetration and antitumor efficacy. Adv Funct Mater.

[110] Kooijmans SAA, Fliervoet LAL, van der Meel R, Fens M, Heijnen HFG, van Bergen En PMP, Henegouwen P, Vader R.M. Schiffelers (2016). PEGylated and targeted extracellular vesicles display enhanced cell specificity and circulation time. J Control Release Off J Control Release Soc.

[111] Nakase I, Futaki S (2015). Combined treatment with a pH-sensitive fusogenic peptide and cationic lipids achieves enhanced cytosolic delivery of exosomes. Sci Rep.

[112] Heusermann W, Hean J, Trojer D, Steib E, von Bueren S, Graff-Meyer A, Genoud C, Martin K, Pizzato N, Voshol J, Morrissey DV, Andaloussi SE, Wood MJ, Meisner-Kober NC (2016). Exosomes surf on filopodia to enter cells at endocytic hot spots, traffic within endosomes, and are targeted to the ER. J Cell Biol.

[113] Friedman JR, Dibenedetto JR, West M, Rowland AA, Voeltz GK (2013). Endoplasmic reticulum-endosome contact increases as endosomes traffic and mature. Mol Biol Cell.

[114] Joshi BS, de Beer MA, Giepmans BNG, Zuhorn IS (2020). Endocytosis of extracellular vesicles and release of their cargo from endosomes. ACS Nano.

[115] Luga V, Zhang L, Viloria-Petit AM, Ogunjimi AA, Inanlou MR, Chiu E, Buchanan M, Hosein AN, Basik M, Wrana JL (2012). Exosomes mediate stromal mobilization of autocrine wnt-PCP signaling in breast cancer cell migration. Cell.

[116] Vitelli R, Santillo M, Lattero D, Chiariello M, Bifulco M, Bruni CB, Bucci C (1997). Role of the small GTPase Rab7 in the late endocytic pathway. J Biol Chem.

[117] Herrmann IK, Wood MJA, Fuhrmann G (2021). Extracellular vesicles as a next-generation drug delivery platform. Nat Nanotechnol.

[118] Vogt S, Bobbili MR, Stadlmayr G, Stadlbauer K, Kjems J, Rüker F, Grillari J, Wozniak-Knopp G (2021). An engineered CD81-based combinatorial library for selecting recombinant binders to cell surface proteins: Laminin binding CD81 enhances cellular uptake of extracellular vesicles. J Extracell Vesicles.

[119] Altei WF, Pachane BC, dos Santos PK, Ribeiro LNM, Sung BH, Weaver AM, Selistre-de-Araújo HS (2020). Inhibition of αvβ3 integrin impairs adhesion and uptake of tumor-derived small extracellular vesicles. Cell Commun Signal.

[120] Nolte-'t Hoen EN, Buschow SI, Anderton SM, Stoorvogel W, Wauben MH (2009). Activated T cells recruit exosomes secreted by dendritic cells via LFA-1. Blood.

[121] El-Sayed A, Harashima H (2013). Endocytosis of gene delivery vectors: from clathrin-dependent to lipid raft-mediated endocytosis. Mol Ther.

[122] Otto GP, Nichols BJ (2011). The roles of flotillin microdomains—endocytosis and beyond. J Cell Sci.

[123] Hao S, Bai O, Li F, Yuan J, Laferte S, Xiang J (2007). Mature dendritic cells pulsed with exosomes stimulate efficient cytotoxic T-lymphocyte responses and antitumour immunity. Immunology.

[124] Feng D, Zhao WL, Ye YY, Bai XC, Liu RQ, Chang LF, Zhou Q, Sui SF (2010). Cellular internalization of exosomes occurs through phagocytosis. Traffic.

[125] Haney MJ, Klyachko NL, Zhao Y, Gupta R, Plotnikova EG, He Z, Patel T, Piroyan A, Sokolsky M, Kabanov AV, Batrakova EV (2015). Exosomes as drug delivery vehicles for Parkinson's disease therapy. J Control Release Off J Control Release Soc.

[126] Tkach M, Kowal J, Zucchetti AE, Enserink L, Jouve M, Lankar D, Saitakis M, Martin-Jaular L, Théry C (2017). Qualitative differences in T-cell activation by dendritic cell-derived extracellular vesicle subtypes. EMBO J..

